# OTME-9. Comprehensive Metabolic Profiling Of high MYC Medulloblastoma Reveals Key Differences Between In *Vitro* And In *Vivo* Glucose And Glutamine Usage

**DOI:** 10.1093/noajnl/vdab070.060

**Published:** 2021-07-05

**Authors:** Khoa Pham, Brad Poore, Allison Hanaford, Micah J Maxwell, Heather Sweeney, Akhila Parthasarathy, Jesse Alt, Rana Rais, Barbara S Slusher, Charles G Eberhart, Eric H Raabe

**Affiliations:** Johns Hopkins University School of Medicine, Baltimore, MD, USA

## Abstract

Reprograming of cellular metabolism is a hallmark of cancer. The metabolic alterations in cancer cells is not only defined by series of genetic mutations, but also reflecting the crosstalk between cancer cells and other factors in the microenvironment. Altering metabolism allows cancer cells to overcome unfavorable conditions, to proliferate and invade. Medulloblastoma is the most common malignant brain tumor of children. Genomic amplification of MYC is a hallmark of a subset of poor-prognosis medulloblastoma. However, the metabolism of high MYC amplified medulloblastoma subgroup remains underexplored. We performed comprehensive metabolic studies of human MYC-amplified medulloblastoma by comparing the metabolic profiles of tumor cells in different environments – in *vitro*, in flank xenografts and in orthotopic xenografts. Principal component analysis showed that the metabolic profiles of brain and flank high-MYC medulloblastoma tumors clustered closely together and separated away from normal brain and the high-MYC medulloblastoma cells in culture. Compared to normal brain, MYC-amplified medulloblastoma orthotopic xenograft tumors showed upregulation of nucleotide, hexosamine biosynthetic pathway (HBP), TCA cycle, and amino acid and glutathione pathways. There was significantly higher glucose up taking and usage in orthotopic xenograft tumor compared to flank xenograft and cells in culture. The data demonstrated that glucose was the main carbon source for the glutamate, glutamine and glutathione synthesis through the TCA cycle. The glutaminase ii pathway was the main pathway utilizing glutamine in MYC-amplified medulloblastoma in vivo. Glutathione was found as the most abundant upregulated metabolite. Glutamine derived glutathione was mainly synthesized through glutamine transaminase K (GTK) enzyme in vivo. In conclusion, we demonstrated that high MYC medulloblastoma adapt to different environments by altering its metabolic pathways despite carrying the same genetic mutations. Glutamine antagonists may have therapeutic applications in human patients.

